# Characterization of Natural Aryl Hydrocarbon Receptor Agonists from Cassia Seed and Rosemary

**DOI:** 10.3390/molecules19044956

**Published:** 2014-04-17

**Authors:** Yoshiaki Amakura, Morio Yoshimura, Masashi Takaoka, Haruka Toda, Tomoaki Tsutsumi, Rieko Matsuda, Reiko Teshima, Masafumi Nakamura, Hiroshi Handa, Takashi Yoshida

**Affiliations:** 1 College of Pharmaceutical Sciences, Matsuyama University; 4-2 Bunkyo-cho, Matsuyama, Ehime 790-8578, Japan; E-Mails: myoshimu@cc.matsuyama-u.ac.jp (M.Y.); matakaoka@shikoku-cc.go.jp (M.T.); aranharu.0205@gmail.com (H.T.); tyoshida@gem.e-catv.ne.jp (T.Y.); 2 Division of Foods, National Institute of Health Sciences; 1-18-1 Kamiyoga, Setagaya-ku, Tokyo 158-8501, Japan; E-Mails: tutumi@nihs.go.jp (T.T.); matsuda@nihs.go.jp (R.M.); rteshima@nihs.go.jp (R.T.); 3 Hiyoshi Corporation, 908 Kitanosho-cho, Omihachiman, Shiga 523-8555, Japan; E-Mails: m.nakamura@hiyoshi-es.co.jp (M.N.); handa@hiyoshi-es.co.jp (H.H.)

**Keywords:** aryl hydrocarbon receptor, health food, cassia seed, rosemary, reporter gene assay

## Abstract

Many recent studies have suggested that activation of the aryl hydrocarbon receptor (AhR) reduces immune responses, thus suppressing allergies and autoimmune diseases. In our continuing study on natural AhR agonists in foods, we examined the influence of 37 health food materials on the AhR using a reporter gene assay, and found that aqueous ethanol extracts of cassia seed and rosemary had particularly high AhR activity. To characterize the AhR-activating substances in these samples, the chemical constituents of the respective extracts were identified. From an active ethyl acetate fraction of the cassia seed extract, eight aromatic compounds were isolated. Among these compounds, aurantio-obtusin, an anthraquinone, elicited marked AhR activation. Chromatographic separation of an active ethyl acetate fraction of the rosemary extract gave nine compounds. Among these compounds, cirsimaritin induced AhR activity at 10–10^2^ μM, and nepitrin and homoplantagenin, which are flavone glucosides, showed marked AhR activation at 10–10^3^ μM.

## 1. Introduction

The aryl hydrocarbon receptor (AhR) is a ligand-dependent transcription factor that is present in mammalian cells and tissues. The AhR has also been referred to as dioxin receptor because it binds environmental pollutants (e.g., dioxins) and is involved in biotoxicity linked to xenobiotic AhR ligand exposure in animals, including cancer, reproductive impairment, and immunological impairment [1,2,3]. Although studies have identified numerous xenobiotic ligands for the AhR, such as dioxins, the essential functions of the AhR are largely unknown; therefore, the AhR is still regarded as an orphan receptor.

Functional elucidation of AhR activation by non-toxic ligands such as food constituents has been reported in recent years [4,5,6]. The AhR has been identified as a target of several signaling pathways that cross-talk with its own regulatory pathway, such as proteasomal degradation, redox-sensitive transcription factors, and mitogen-activated protein kinases (MAPKs) [7,8]. Several studies have also found that the AhR plays an important role in immune system function [9,10,11,12]. For example, activation of the AhR is associated with various effects on dendritic cells (DCs) and regulatory T cells and has been shown to mediate the Th1/Th2 cell balance. These cells play a major role in the development of food allergies, an increasing health problem in both humans and animals. Despite existing knowledge regarding the risk factors of and cellular mechanisms underlying food allergies, no approved treatments are yet available. Activation of the AhR by dioxin-like compounds has been shown to suppress allergic sensitization by reducing the absolute number of precursor and effector T cells, preserving CD4^+^ CD25^+^ Foxp^3+^ T_reg_ cells, and affecting DCs and their interactions with effector T cells. Additionally, tranilast, an anti-allergy drug, has been shown to cause significant upregulation of *microRNA* (*miR*)-*302* by activation of the AhR [13]. Thus, dietary ligands of the AhR may have anti-inflammatory, anti-allergy, anti-cancer, and immunoregulatory effects. However, while although the role of the AhR in the response to environmental toxins is widely accepted, its broader role in adapting the response to natural ligands is limited. Therefore, it is necessary to characterize various natural AhR ligands.

In the current study, we sought to further characterize AhR agonists present in foods. We examined the AhR activities of 37 health food materials using an *in vitro* reporter gene assay called the chemical-activated luciferase gene expression (CALUX) assay [14,15,16]. Active sample extracts were subsequently fractionated, and chromatography was performed to characterize the fractions containing AhR activity and associated individual constituents.

## 2. Results and Discussion

### 2.1. AhR Activities of Health Food Materials

**Figure 1:**
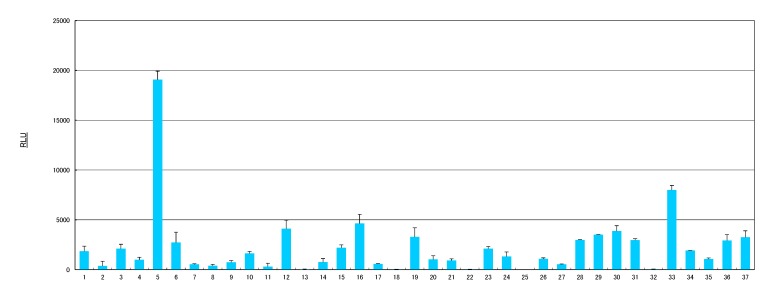
Induction of luciferase activity by health food materials in the CALUX assay. The numbers on the x-axis describe the components listed in Table 1. Sample extracts were used at a final concentration of 100 μg/mL. Results are expressed as means ± SDs.

The *in vitro* AhR activation potencies of 37 samples, including the fruits and herbs listed in Table 1, were estimated using the CALUX assay, and the results are shown in Figure 1. Of the samples tested, sample 5 (cassia seed extract) showed the most remarkable induction of luciferase activity, followed by sample 33 (rosemary extract), with luciferase activity producing more 8,000 relative light units (RLU). Samples 12 (*Eleutherococcus senticosus* rhizome), 16 (fenugreek), 19 (giant crape-myrtle), 29 (parsley), 30 (perilla herb), and 37 (yarrow) also exhibited luciferase activity higher than 3,000 RLU. The data suggest that cassia seed and rosemary may contain significant natural AhR agonists.

**Table 1:** List of health food materials used for the estimation of AhR activity

No.	Materials
1	Ashitaba (Japanese name) (*Angelica keiskei*)
2	Aloe (*Aloe arborescens*)
3	Amachazuru (Japanese name) (*Gynostemma pentaphyllum*)
4	Bitter melon (*Momordica charantia*)
5	Cassia seed (*Cassia obtusifolia*)
6	Celery (*Apium graveolens*)
7	Coix seed (*Coix lacryma-jobi*)
8	Cornus fruit (Cornus officinalis)
9	Crataegus fruit (*Crataegus cuneata*)
10	Echinacea (*Echinacea purpurea*)
11	Elder (*Sambucus racemosa*)
12	*Eleutherococcus senticosus* rhizome (*Eleutherococcus senticosus*)
13	Eucalyptus leaf (*Eucalyptus globulus*)
14	Eucommia bark (*Eucommia ulmoides*)
15	Fennel (*Foeniculum vulgare*)
16	Fenugreek (*Trigonella foenum-graecum*)
17	Field horsetail (*Equisetum arvense*)
18	Garcinia (*Garcinia verrucosa*)
19	Giant crape-myrtle (*Lagerstroemia speciosa*)
20	Ginger (*Zingiber officinale*)
21	Ginkgo (*Ginkgo bilob*a)
22	Gymnema (*Gymnema sylvestre*)
23	Kaki persimmon (*Diospyros kaki*)
24	Lemon balm (*Melissa officinalis*)
25	Lemon grass (*Cymbopogon citratus*)
26	Linden (*Tilia europaea*)
27	Maca (*Lepidium meyenii*)
28	Mugwort (*Artemisia indica*)
29	Parsley (*Petroselinum crispum*)
30	Perilla herb (*Perilla frutescens*)
31	Plantago herb (*Plantago asiatica*)
32	Rabdosia herba (*Rabdosia japonica*)
33	Rosemary (*Rosmarinus officinalis*)
34	Sesame (*Sesamum indicum*)
35	Star anise (*Illicium verum*)
36	Sweet hydrangea leaf (*Hydrangea macrophylla*)
37	Yarrow (*Achillea millefolium*)

### 2.2. Identification and AhR Activity of Constituents

**Figure 2:**
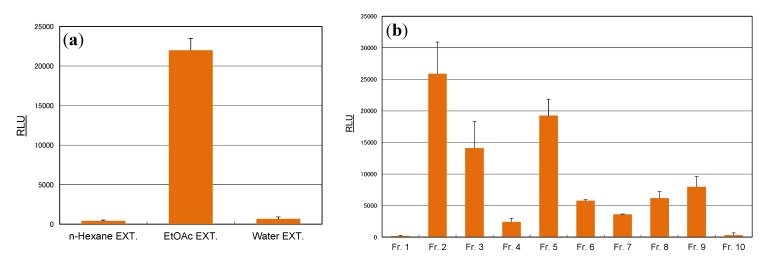
Induction of luciferase activity by cassia seed extracts in the CALUX assay. (**a**) Extracts from cassia seed. (**b**) Fractions from ethyl acetate extracts. Sample extracts were used at a final concentration of 100 μg/mL. Results are expressed as means ± SDs.

To characterize the active components in sample 5 (cassia seed extract), the extract was first partitioned with organic solvent for separation into *n*-hexane-, ethyl acetate-, and water-soluble fractions. As shown in Figure 2a, AhR activity was present only in the ethyl acetate extract, which was separated by chromatography over Sephadex LH-20 with ethanol to afford 10 fractions (Frs. 1–10).

**Figure 3:**
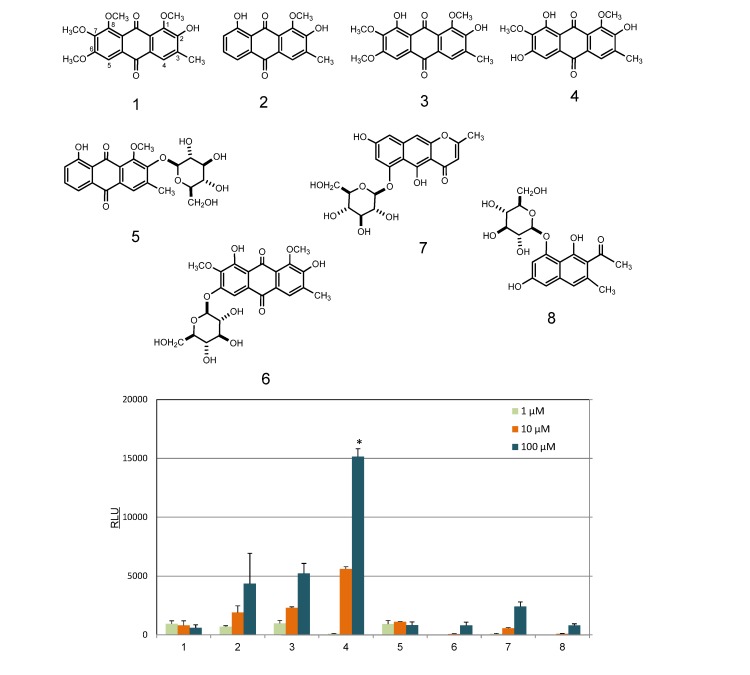
Induction of luciferase activity in the CALUX assay of compounds isolated from cassia seeds. **1**, chryso-obtusin; **2**. obtusifolin; **3**. obtusin; **4**. aurantio-obtusin; **5**. obtusin 2-*O*-glucoside; **6**. aurantio-obtusin 6-*O*-glucoside; **7**. nor-rubrofusarin 6-*O*-glucoside; **8**. 6-hydroxymusizin 8-*O*-glucoside. * *p* < 0.05 *vs.* IAA.

Fractions 2, 3, and 5, which exhibited marked AhR activation (Figure 2b), were purified by preparative TLC to afford eight compounds: chryso-obtusin (**1**), obtusifolin (**2**), obtusin (**3**), aurantio-obtusin (**4**), obtusin 2-*O*-glucoside (**5**), aurantio-obtusin 6-*O*-glucoside (**6**), nor-rubrofusarin 6-*O*-glucoside (**7**), and 6-hydroxymusizin 8-*O*-glucoside (**8**). Among these isolates, aurantio-obtusin (**4**) elicited marked AhR activation, followed by obtusifolin (**2**) and obtusin (**3**). In contrast, the glycosides [obtusifolin 2-*O*-glucoside (**5**), aurantio-obtusin 6-*O*-glucoside (**6**), nor-rubrofusarin 6-*O*-glucoside (**7**), and 6-hydroxymusizin 8-*O*-glucoside (**8**)] showed only slight activation of AhR (Figure 3). The influence of this glycosidic feature on the activity of the related anthraquinones was similar to our previous findings that the AhR activity of isoflavones tended to be weakened by glycosidation [4]. It is notable that the presence of a hydroxyl group at C-8 on the anthraquinone skeleton is necessary for AhR activation.

Additionally, aurantio-obtusin (**4**), which was the most active compound, had a hydroxyl group at C-7 and C-9, which may also contribute to AhR activation. However, to discuss the structure-activity relationships in anthraquinones, additional data from more compounds are required. The results of the present study revealed that AhR activation by the cassia seed extract is associated with anthraquinones and that aurantio-obtusin (**4**) may be an important natural AhR agonist.

**Figure 4:**
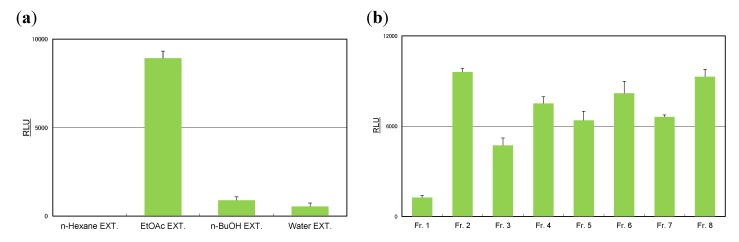
Induction of luciferase activity by rosemary extracts in the CALUX assay. (**a**) Extracts from rosemary. (**b**) Fractions from ethyl acetate extracts. Sample extracts were used at a final concentration of 100 μg/mL. Results are expressed as means ± SDs.

**Figure 5:**
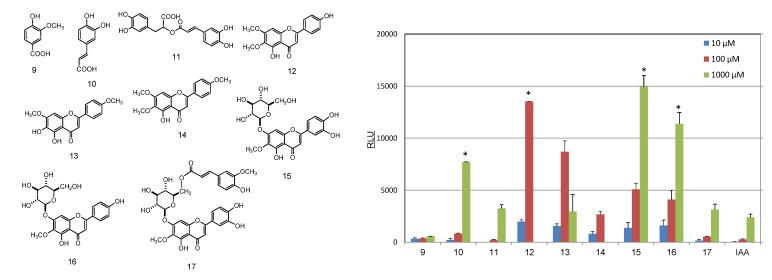
Induction of luciferase activity by compounds isolated from rosemary. The CALUX assay was used to measure luciferase activity. **9**. vanillic acid; **10**. caffeic acid; **11**. rosmarinic acid; **12**. cirsimaritin; **13**. ladanein; **14**. salvigenin, **15**: nepitrin, **16**. homoplantaginin; **17**. 6ʺ-*O*-(*E*)-feruloylnepitrin; IAA. indole 3-acetic acid. * *p* < 0.05 *vs.* IAA.

For the rosemary extract, AhR activation was also shown by the ethyl acetate-soluble fraction (Figure 4a). To identify the active compounds present, the ethyl acetate extract was subjected to chromatographic purification and chromatographed over a Sephadex LH-20 column with ethanol to afford eight fractions (Frs. 1–8). Fractions 2–8, which exhibited marked AhR activation (Figure 4b), were purified using a MCI-gel CHP-20P and YMC gel ODS-AQ column to give rosmarinic acid (**11**) as a major component and other eight compounds, *i.e.*, vanillic acid (**9**), caffeic acid (**10**), cirsimaritin (**12**), ladanein (**13**), salvigenin (**14**), nepitrin (**15**), homoplantaginin (**16**), and 6ʺ-*O*-(*E*)-feruloylnepitrin (**17**), as UV-sensitive constituents (Figure 5).

The ability of compounds **9**–**15**, isolated from rosemary extract, to activate the AhR were examined using reporter gene assays. As shown in Figure 5, cirsimaritin (**12**) and ladanein (**13**) exhibited significant AhR activation at 10–10^2^ μM. In contrast, compounds **12**–**14** induced cell death at 10^3^ μM (Figure 5). Moreover, nepitrin (**15**) and homoplantagenin (**16**), which are flavone glucosides, showed marked AhR-binding activity at concentrations ranging from 10–10^3^ μM lower than those required for binding by indole 3-acetic acid (IAA), a typical natural AhR ligand [8].

As mentioned earlier, AhR activation tends to be weakened by glycosidation of the parent AhR ligand. This tendency has been observed even for flavonoid ligands [4]. In the present study, nepitrin (**15**) and homoplantagenin (**16**), which are flavone glucosides, were found to have noticeable AhR activity.

Some compounds characterized as potential AhR agonist candidates in the current study have been reported to have various biological functions beneficial to human health. For example, lipolytic, antilipogenic, and antiproliferative activities have been identified as biological properties of cirsimaritin (**14**) [17], and nepitrin (**15**) has been reported to have anti-inflammatory and gastroprotective activity [18,19]. Recently, several studies have reported that activation of AhR may be involved in various immune responses as described above; therefore, natural AhR ligands are expected to have beneficial regulatory roles in humans, mediating anti-allergy and anti-cancer effects. Further studies on AhR-activating ingredients derived from natural foods may clarify both the physiological significance of the AhR and the benefits derived from food constituents.

## 3. Experimental

### 3.1. General

^1^H- and ^13^C-NMR spectra (500 MHz for ^1^H and 126 MHz for ^13^C) were recorded on a Bruker AVANCE 500 instrument (Bruker BioSpin, Billerica, MA, USA), and chemical shifts are given in ppm values relative to those of the solvents [chloroform-*d* (δ_H_ 7.26; δ_C_ 77.16), methanol-*d*_4_ (δ_H_ 3.30; δ_C_ 49.0), dimethylsulfoxide (DMSO)-*d*_6_ (δ_H_ 2.50; δ_C_ 39.5), and acetone-*d*_6_ (δ_H_ 2.04; δ_C_ 49.0)] on a tetramethylsilane scale. The standard pulse sequences programmed for the instrument (AVANCE 500) were used for each 2D measurement (COSY, HSQC, and HMBC). *J*_CH_ was set at 10 Hz in HMBC. Electrospray ionization (ESI)-MS, and high-resolution (HR) ESI-MS spectra were obtained using a micrOTOF-Q (Bruker Daltonics, Billerica, MA, USA) mass spectrometer with acetonitrile as the solvent. UV spectra were recorded on a Shimadzu UVmini-1240 system (Shimadzu, Kyoto, Japan).

The reversed-phase (RP) HPLC conditions were as follows: column, L-column ODS (5 μm, 150 × 2.1 mm i.d.) (Chemicals Evaluation and Research Institute, Tokyo, Japan); mobile phase, 5% acetic acid (solvent A) and acetonitrile (solvent B) (0–30 min, 0%–50% B in A; 30–35 min, 50%–85% B in A; 35–40 min, 85%–85% B in A); injection volume, 2 μL; column temperature, 40 °C; flow rate, 0.3 mL/min; and detection, 200–400 nm. TLC was performed on Silica Gel 60 F_254_ plates (Merck, Darmstadt, Germany), and the spots were visualized under a UV lamp (254 nm). Column chromatography was conducted using Sephadex LH-20 (GE Healthcare, Little Chalfont, England), MCI Gel CHP-20P (75–150 μm) (Mitsubishi Chemical Co., Tokyo, Japan), YMC GEL ODS-AQ (AQ12S50) (YMC Co., Ltd., Kyoto, Japan), and Silica Gel 60 (Nacalai Tesque, Kyoto, Japan) columns.

### 3.2. Samples and Reagents

The reagents used in the present study were purchased from Wako Pure Chemical Industries, Ltd. (Osaka, Japan) and Nacalai Tesque, and 37 health food materials, as shown in Table 1, were obtained from Uchida Wakanyaku Ltd. (Tokyo, Japan), Tochimoto Tenkaido Ltd. (Osaka, Japan), and Nagaoka Perfumery Ltd. (Osaka, Japan). The species were identified by the Herbarium of the College of Pharmaceutical Sciences, Matsuyama University, where the voucher specimens were deposited. All other chemicals were of analytical reagent grade.

### 3.3. Extraction

The health food samples were prepared as follows: The materials (1 g) were homogenized in aqueous ethanol [ethanol/water (4:1)] (30 mL) for 10 min and filtered. The filtrates were concentrated under reduced pressure and freeze-dried.

### 3.4. Isolation of Compounds from Cassia Seeds

Cassia seeds (400 g) purchased from Uchida Wakanyaku Ltd. were homogenized in 80% ethanol [ethanol/H_2_O (8:3)] (4 L), and a concentrated solution (*ca*. 0.15 L) was extracted successively with *n*-hexane (0.45 L) and ethyl acetate (0.45 L) to obtain the respective *n*-hexane (6.14 g), ethyl acetate (1.54 g), and water (34.47 g) extracts.

The ethyl acetate extract (0.7 g) was chromatographed over a Sephadex LH-20 column with ethanol to give 10 fractions (Frs. 1–10). Frs. 2 and 3 (50 mg) were subjected to preparative TLC [ethyl acetate/methanol (3:1), *n*-hexane/ethyl acetate/acetic acid (10:5:2), and then chloroform/methanol (95:5)] to give chryso-obtusin (**1**) (2 mg), obtusifolin (**2**) (2 mg), obtusin (**3**) (2 mg), aurantio-obtusin (**4**) (2 mg), and obtusin 2-*O*-glucoside (**5**) (2.6 mg). Fr. 5 (100 mg) was similarly purified with preparative TLC [chloroform/methanol/H_2_O (14:6:1)] to afford aurantio-obtusin 6-*O*-glucoside (**6**) (2.7 mg), nor-rubrofusarin 6-*O*-glucoside (**7**) (11 mg), and 6-hydroxymusizin 8-*O*-glucoside (**8**) (2.1 mg). Fr. 4 (180 mg) was subjected to column chromatography over silica gel 60 (ϕ 2.0 × 20 cm) with chloroform/methanol (9:1) to give obtusin 2-*O*-glucoside (**5**) (4.1 mg). These known compounds were identified by direct comparison with valid standards or by comparison of their spectral data with those reported in the literature [20,21].

### 3.5. Isolation of Compounds from Rosemary

Rosemary leaves (526 g) provided by Nagaoka Perfumery Co. Ltd. were homogenized in 80% ethanol (ethanol/H_2_O 8:2) (5 L), and a concentrated solution (*ca*. 0.15 L) was extracted successively with *n*-hexane (4 L), ethyl acetate (4 L), and *n*-butanol (4 L) to give the respective *n*-hexane (6.14 g), ethyl acetate (1.54 g), *n*-butanol (14.84 g), and water (34.47 g) extracts. The ethyl acetate extract (1 g) was chromatographed over Sephadex LH-20 with ethanol to give eight fractions (Frs.1–8). Frs. 2–8 (876 mg in total) were combined and further subjected to column chromatography over YMC GEL ODS-AQ and MCI Gel CHP-20P columns with aqueous methanol to yield vanillic acid (**9**) (2 mg), caffeic acid (**10**) (2 mg), rosmarinic acid (**11**) (82.8 mg), cirsimaritin (**12**) (2 mg), ladanein (**13**) (2 mg), salvigenin (**14**) (1.5 mg), nepitrin (**15**) (13.5 mg), homoplantaginin (**16**) (6 mg), and 6ʺ-*O*-(*E*)-feruloylnepitrin (**17**) (2 mg). These compounds were identified by direct comparison with authentic specimens or by comparison of their spectral data with those reported in the literature [22,23,24,25].

### 3.6. Estimation of AhR Ligand Activity

The extracts and compounds were dissolved in DMSO and evaluated for AhR-binding activity using a luciferase assay (CALUX assay). The CALUX assay for AhR ligand activity was conducted as follows. Mouse hepatoma H1L1 cells (*ca*. 1.5 × 10^5^ cells/well) were cultured in 96-well culture plates, and the samples were dissolved in DMSO and then added at final concentrations of 1–10^2^ μg/mL (or μM in compound)] in three steps in fractions. The final DMSO concentration was 1% in the cell culture medium. The plates were incubated at 37 °C in 5% CO_2_ for 24 h for optimal expression of luciferase activity. After incubation, cell viability was confirmed using a microscope. Subsequently, the medium was removed and the cells were lysed. After addition of luciferin as the substrate, luciferase activity was determined using a luminometer (Centro LB960; Berthold, Bad Wildbad, Germany) and recorded as RLUs. The values represent the mean ± SD of at least two or three independent determinations for each experiment. Statistical significance was analysed using the Student’s *t* test.

## 4. Conclusions

In this study, we examined the effects of 37 health food materials on AhR activity using a reporter gene assay and found that cassia seed and rosemary extracts elicited notable AhR activation. To characterize the AhR-activating substances within these extracts, the respective extracts were subjected to fractionation followed by estimation of AhR activity. Eight compounds were isolated and identified from the active fractions of the cassia seed extract. Among them, aurantio-obtusin (**4**), an anthraquinone, was characterized as an effective AhR-activating ligand. In rosemary, nine compounds were isolated from the active extract. Nepitrin (**15**) and homoplantagenin (**16**), which are flavone glucosides, showed marked AhR-binding activity.
